# Multum in Parvo

**Published:** 1897-04

**Authors:** 


### Multum in Parvo.

A young lady of very extraordinary capacity lately addressed the following letter to her cousin: "We is all well; and mother's got the his Terrix; brother Tom has got the Hupin Kaugh; and sister Ann has got a babee; and I hope these few lines will find you the same. Rite sune. Your aphectionate kuzzen."—*Tit-bits.*

